# Competition from native hydrophytes reduces establishment and growth of invasive dense-flowered cordgrass (*Spartina densiflora*)

**DOI:** 10.7717/peerj.1260

**Published:** 2015-10-08

**Authors:** Ahmed M. Abbas, Adam M. Lambert, Alfredo E. Rubio-Casal, Alfonso De Cires, Enrique M. Figueroa, Jesús M. Castillo

**Affiliations:** 1 Department of Botany, University of South Valley, Qena, Egypt; 2 Marine Science Institute, University of California, Santa Barbara, CA, United States; 3 Department of Plant Biology and Ecology, University of Sevilla, Sevilla, Spain

**Keywords:** Brackish marshes, Inter-specific competition, Invasion, Salt marshes, *Phragmites australis*, Intra-specific competition, *Typha domingensis*

## Abstract

Experimental studies to determine the nature of ecological interactions between invasive and native species are necessary for conserving and restoring native species in impacted habitats. Theory predicts that species boundaries along environmental gradients are determined by physical factors in stressful environments and by competitive ability in benign environments, but little is known about the mechanisms by which hydrophytes exclude halophytes and the life history stage at which these mechanisms are able to operate. The ongoing invasion of the South American *Spartina densiflora* in European marshes is causing concern about potential impacts to native plants along the marsh salinity gradient, offering an opportunity to evaluate the mechanisms by which native hydrophytes may limit, or even prevent, the expansion of invasive halophytes. Our study compared *S. densiflora* seedling establishment with and without competition with *Phragmites australis* and *Typha domingensis*, two hydrophytes differing in clonal architecture. We hypothesized that seedlings of the stress tolerant *S. densiflora* would be out-competed by stands of *P. australis* and *T. domingensis*. Growth, survivorship, biomass patterns and foliar nutrient content were recorded in a common garden experiment to determine the effect of mature *P. australis* and *T. domingensis* on the growth and colonization of *S. densiflora* under fresh water conditions where invasion events are likely to occur. Mature *P. australis* stands prevented establishment of *S. densiflora* seedlings and *T. domingensis* reduced *S. densiflora* establishment by 38%. Seedlings grown with *P. australis* produced fewer than five short shoots and all plants died after ca. 2 yrs. Our results showed that direct competition, most likely for subterranean resources, was responsible for decreased growth rate and survivorship of *S. densiflora*. The presence of healthy stands of *P. australis*, and to some extent *T. domingensis*, along river channels and in brackish marshes may prevent the invasion of *S. densiflora* by stopping the establishment of its seedlings.

## Introduction

Competition between native and invasive plant species has been broadly studied in marshes (Ungar, 1998), however experimental studies to determine the nature of ecological interactions between invasive and native species are necessary for conserving and restoring native species in impacted habitats (Parker, Simberloff & Lonsdale, 1999; Byers & Goldwasser, 2001).

Theory predicts that species boundaries along environmental gradients are determined by physical factors in stressful environments and by competitive ability in benign environments (Crain & Bertness, 2006; Maestre et al., 2009; Engels, Rink & Jensen, 2011). Engels & Jensen (2010) showed that such a relationship controls plant zonation in estuaries. Plants transplanted from low salinity environments (hydrophytes) to salt marshes performed poorly regardless of whether neighbouring vegetation was present or not, and conversely, plants growing in high salinities (halophytes) had low biomass and high mortality rates in the presence of neighbors when transplanted to freshwater marshes. Without neighbors, biomass of halophytes in freshwater wetlands was similar to or higher than that in salt marshes. These results showed a shift in the importance of competition along the estuarine salinity gradient. Still, little is known about competitive outcomes among native and invasive plants differing in salinity tolerance or the life history stage at which these mechanisms operate—information central to managing plant invasions in coastal and estuarine environments.

The South American cordgrass, *S. densiflora* Brongn. (Poaceae), is a clonal plant invading estuaries in Europe (Nieva et al., 2001) and North America (Kittelson & Boyd, 1997), but the impacts to these systems and mode of invasion are poorly known. In Europe, *S. densiflora* invades a wide range of habitats, including brackish marshes and river banks (Nieva et al., 2001; Curado et al., 2010) and is interacting with native plants over a strong salinity gradient that may influence competition among species. The native congener *S. maritima* Curtis (Fernald) may be succumbing to *S. densiflora* invasion at middle marsh elevations (Castillo et al., 2008; Castillo & Figueroa, 2009), but invasion has not occurred in intact stands of indigenous freshwater and brackish marsh hydrophytes, such as the clonal dominant wetland plants *Typha domingensis* Pers. (southern cattail) and *Phragmites australis* (Cav.) Trin. ex Steud. (common reed). *Spartina densiflora* invades bare sediments in new areas by seedlings established from numerous seeds dispersed by water (Kittelson & Boyd, 1997; Nieva et al., 2001). Thus, *S. densiflora* invasion offers an opportunity to analyze the mechanisms by which native hydrophytes would limit the expansion of invasive halophytes in mid to high marsh habitats.

Our study compared invasive *S. densiflora* seedling establishment with and without competition with native *P. australis* and *T. domingensis*, two hydrophytes with differing clonal architecture: *P. australis* has a high stem density with narrow stem diameters and *T. domingensis* has a lower stem density, but thicker diameter stems. We hypothesized that seedlings of the stress tolerant *S. densiflora* would be out-competed by mature stands of *P. australis* and *T. domingensis* under low salinity conditions following the general ecological theory that stress tolerant plants have a lower competitive capacity than stress-intolerant but fast-growing plants. We compared growth, survivorship, biomass allocation patterns and foliar nutrient content of *S. densiflora* seedlings in response to inter- and intra-specific competition and in the absence of competition to explore the ability of native *P. australis* and *T. domingensis* to prevent *S. densiflora* invasion under low salinity conditions and at early life-stages of the invasion process.

## Materials and Methods

### Experimental design

*Phragmites australis* and *Typha domingensis* rhizomes and *S. densiflora* seeds were collected in Odiel Marshes (Southwest Iberian Peninsula). *Phragmites australis* and *T. domingensis* rhizomes were planted and grown in peat soil in plastic pots (12 cm diameter and 15 cm height; volume of 2.75 l) until they established mature stands with similar densities to those found in wetlands in the Southwest Iberian Peninsula. *Spartina densiflora* seedlings were obtained for experiments from seeds sown on peat soil in flats in the greenhouse. Seeds and seedlings were watered regularly to maintain moist soils until transplanted into treatments.

The common garden experiment was initiated in January 2008 and conducted over two years in a common garden at the University of Seville, Spain. Four treatments (two interspecific competition treatments, one intraspecific competition treatment, and one no competition treatment) were established using transplanted *S. densiflora* seedlings of similar size: (1) Five seedlings of *S. densiflora* transplanted into a pot containing an established *P. australis* stand (*n* = 6 pots); (2) Five seeds of *S. densiflora* transplanted into a pot containing an established *T. domingensis* stand (*n* = 10 pots); (3) Five seedlings of *S. densiflora* transplanted into a pot containing an established *S. densiflora* stand (*n* = 5 pots); and (4) one seedling of *S. densiflora* without intra- and inter-specific competition (*n* = 5 pots). *S. densiflora* seedlings were placed at a depth of 0.5 cm; one at the centre of the pot and the other four around it spaced 3 cm apart. Shoot density and height, and above-ground biomass (AGB) and below-ground biomass (BGB) of *P. australis* and *T. domingensis* used in our experiment were within the range of those reported previously in field studies (Sobrero, Sabbatini & Fernandez, 1997; Sharma et al., 2008; Engloner, 2009). Initial planting conditions imitated natural conditions during autumn-winter periods in Southwest Iberian Peninsula when *S. densiflora* seeds germinate inside native stands with most of their aerial biomass senesced. Plants were maintained at ambient light and temperature and watered daily with fresh water, and pots were placed in pools keeping their base permanently flooded to a height of 3 cm.

### Abiotic environment

Light intensity, and soil salinity (measured as electrical conductivity), pH, and redox potential were measured for each pot to evaluate if biotic interactions among species changed the abiotic environment. Photosynthetic photon flux density (PPFD) was recorded with a portable photometer (LI-COR Instruments, Inc., Lincoln, Nebraska, USA) at ground level during midday outside and within the stands of *P. australis* and *T. domingensis* during summertime (in July 2008) coinciding with maximum biomass accumulation. At the end of the experiment, soil samples were collected from each pot, dried at 60 °C for two days, and then sieved through mesh to remove particles greater than 2 mm. Total soluble salt concentration (salinity) was determined by measuring electrical conductivity (Rhoades, 1996). To determine electrical conductivity, 60 ml of 0.1 M calcium chloride was added to 20 g of soil (3:1 mixture) and mixed on an orbit shaker for 30 min. Conductivity was measured at 21.0 ± 0.5 °C using a CM35+ meter (Crison Instruments, Inc., Barcelona, Spain). To determine soil pH, 30 g of soil was mixed with 30 ml deionized water (1:1 mixture) and mixed on an orbit shaker for 30 min. pH was measured with a CM35+ meter. Redox potential of the soil between 0–5 cm deep was determined with a portable meter and electrode system (Crison Instruments, Inc., Barcelona, Spain).

### Survivorship, shoot production, height and biomass

The number of live *S. densiflora* seedlings, and live *P. australis*, *T. domingensis*, and *S. densiflora* shoots were counted periodically from the beginning of the experiment. Shoot height of *Spartina densiflora* seedlings was measured from the base of the shoot to the tip of the longest leaf (*n* = 5–10 pots; 5 shoots of different clones, or per pot).

At the end of the experiment (February 2010), AGB and BGB were recorded for every clone of each species in each treatment (*n* = 5–10 pots; 5–50 clones per species and treatment). Stems of all plants were harvested, dried at 80 °C for 48 h, and weighed. AGB was divided into dead and live shoots and leaves, and BGB was divided into rhizomes and roots. AGB and BGB for *P. australis* and *T. domingensis* stands were also recorded at the beginning of the experiment using extra pots maintained under experimental conditions.

### Leaf nitrogen and carbon content

Total leaf carbon (C) and nitrogen (N) content were determined for the three plant species in July 2008, when *Spartina* seedlings were large enough to contain enough leaf tissue for these analyses. Three leaves per clone within each pot, and for each treatment, were collected and pooled for analysis. The samples were dried in an oven at 80 °C for 48 h, pulverized using a grinder (Cyclotech, Inc., Cypress, California, USA) and filtered using a screen of 80-µm. Total C and N concentration was determined for undigested samples with an elemental analyzer (Leco Instruments, Inc., Saint Joseph, Michigan, USA).

### Statistical analysis

Analyses were conducted using SPSS release 12.0 (SPSS Inc., Chicago, IL). Data were tested for normality with the Kolmogorov–Smirnov test and for homogeneity of variance with the Levene test at *P* > 0.05. When homogeneity of variance between groups was not found, data were transformed using the following functions: ln(*x*), 1/*x* and √*x*. Analysis of variance was used to detect differences in the response variables among competition treatments and Tukey’s Honest Significant Difference (HSD) test was used to detect differences among treatments only if *F*-test was significant at *P* = 0.05. Student’s *t*-test for independent samples was applied to compare AGB and BGB between *T. domingensis* and *P. australis*. Deviations were calculated as the standard error of the mean.

## Results

### Abiotic environment

Ambient Photosynthetic Photon Flux Density (PPFD) measured outside pots with native plant stands averaged 1,660 ± 105 µmol photon m^−2^ s^−1^ at full sunlight. PPFD measured within P. australis stands was 55% (928 ± 206 µmol photon m^−2^ s^−1^) of full light and was not significantly different than PPFD within T. domingensis stands, which was 63% of full light (1,057 ± 151 µmol photon m^−2^ s^−1^; *t*-test, >0.05). Soil electrical conductivity, pH, and redox potential were not significantly different among treatments (*P* > 0.05). Mean soil pH was ca. 6 and conductivity varied between 0.30 and 2.32 mS cm^−1^. Soil redox potential was always positive (ca. +130 mV).

### Survivorship, shoot production and height

**Figure 1:**
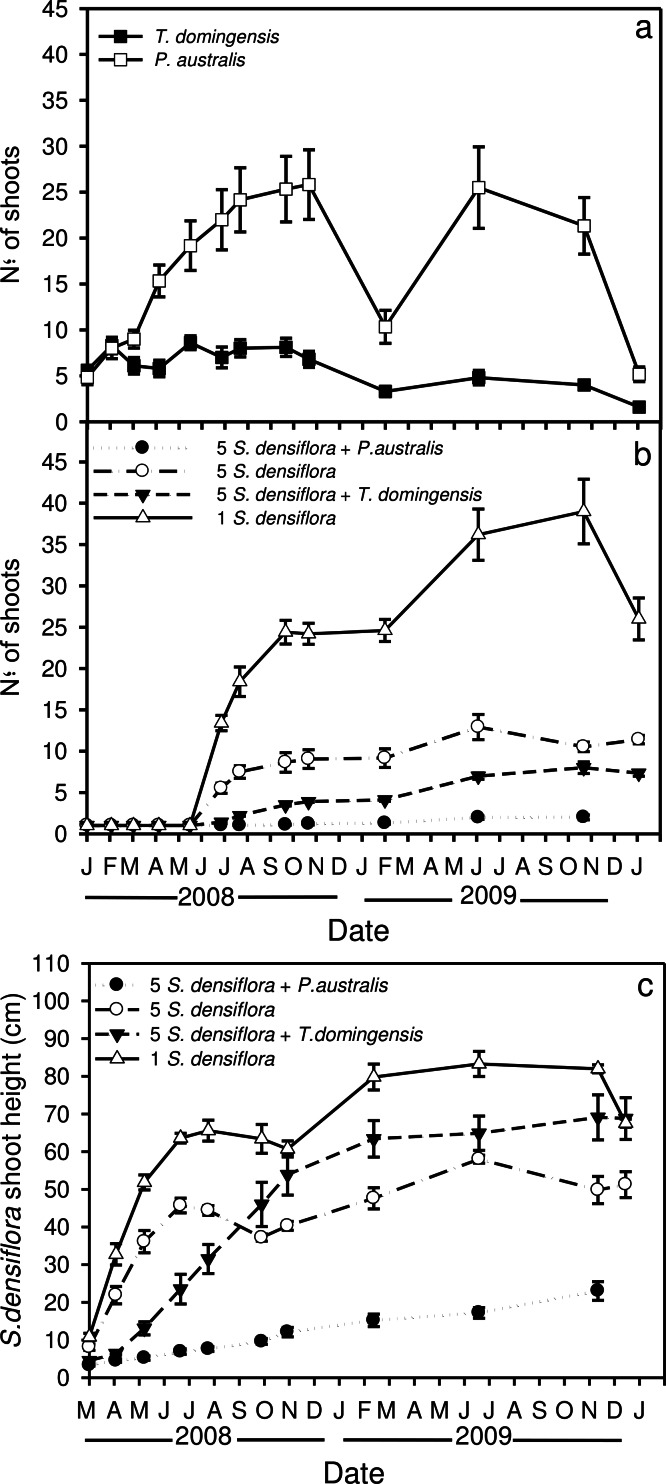
Number of live shoots over time. Temporal variation of the number of live shoots (A) for *Phragmites australis*, *Typha domingensis* and (B) *S. densiflora*, and (C) *S. densiflora* shoot height (cm)for four treatments: 5 *Spartina* seedlings within *Phragmites australis* or within *Typha domingensis*, 5 *Spartina* seedlings and 1 *Spartina* seedling.

*Phragmites australis* consistently had a higher shoot density than *T. domingensis* over the course of the experiment (Fig. 1). Shoot senescence increased over the winter for both native species, but was higher for *P. australis*.

Competition treatments during the first 135 days had no effect on the number of live seedlings of *S. densiflora*. But, by the end of the experiment (ca. two years), no *S. densiflora* seedlings planted into *P. australis* stands survived. Seedlings growing within *T. domingensis* stands were less impacted, having a survival rate of 62%. In contrast, all seedlings planted alone or with other *Spartina* seedlings survived.

Competitive interaction of *Spartina* seedlings with both native species caused depressed development, resulting in fewer and short shoots, and lower biomass than in the absence of competition. Average shoot density of *S. densiflora* seedlings growing alone was 26 ± 3 shoots clone^−1^, followed by the *S. densiflora* monoculture with intra-specific competition (11 ± 1 shoots clone^−1^), seedlings growing within *T. domingensis* (ca. 7 ± 0 shoots clone^−1^), and seedlings growing with *P. australis*(with 2 ± 0 live shoots clone^−1^ just before dying) (*P* < 0.05) (Fig. 1). At the end of the experiment, the shortest *S. densiflora* seedlings were those that had died within *P. australis* stands and averaged 23.0 ± 2.5 cm, followed by those growing in monoculture (51.3 ± 3.4 cm) and by those growing alone or within *T. domingensis* stands (68.8 ± 5.5 cm tall) (*P* < 0.05) (Fig. 1).

### Biomass

**Figure 2:**
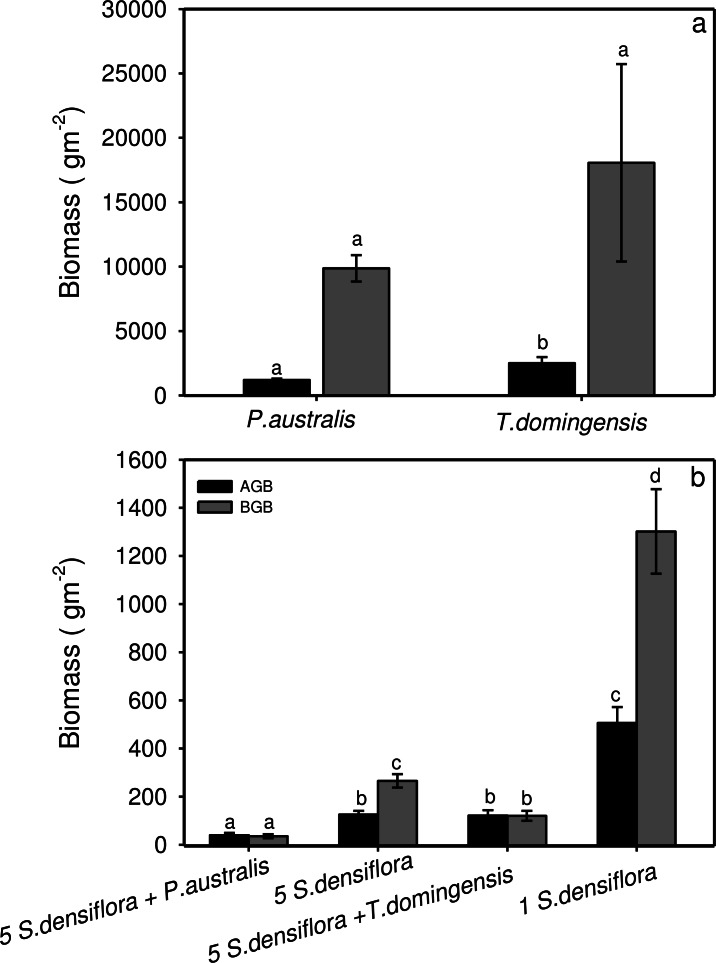
Above- and below-ground biomass for *Phragmites australis* and *Typha domingensis* stands, and *S. densiflora* seedlings in four different competition treatments. Above- (AGB; black bars) and below-ground (BGB; gray bars) biomass (g m^−2^) for (A) *Phragmites australis* and *Typha domingensis* stands and (B) for *S. densiflora* seedlings growing in four different competition treatments Different letters indicate significant difference among treatments (ANOVA or *t*-test, *P* < 0.05).

Both *P. australis* and *T. domingensis* had lower allocation to above-ground than to below-ground structures, but *T. domingensis* did have a higher AGB (ca. 2,500 g m^−2^) than *P. australis* (ca. 1,200 g m^−2^) at the end of the experiment (*t*-test, *P* < 0.05). BGB of *T. domingensis* and *P. australis* stands was similar and averaged ca. 14,000 g m^−2^ for each species (*t*-test, *P* > 0.05) (Fig. 2).

At the end of the experiment, AGB and BGB of *Spartina* seedlings growing alone were significantly higher (AGB: 500 ± 66 g m^−2^; BGB: 1,300 ± 175 g m^−2^) than for the other treatments (AGB: ca. 125 g m^−2^; BGB: ca. 200 g m^−2^) (AGB: *P* < 0.05; BGB: *P* < 0.05). In addition, *S. densiflora* seedlings growing in the intraspecific competition treatment had greater BGB than those growing within *T. domingensis* stands. *S. densiflora* seedlings growing within *P. australis* had the lowest AGB (40 ± 9 g m^−2^) and BGB (36 ± 8 g m^−2^; Fig. 2).

### Leaf nitrogen and carbon content

**Figure 3:**
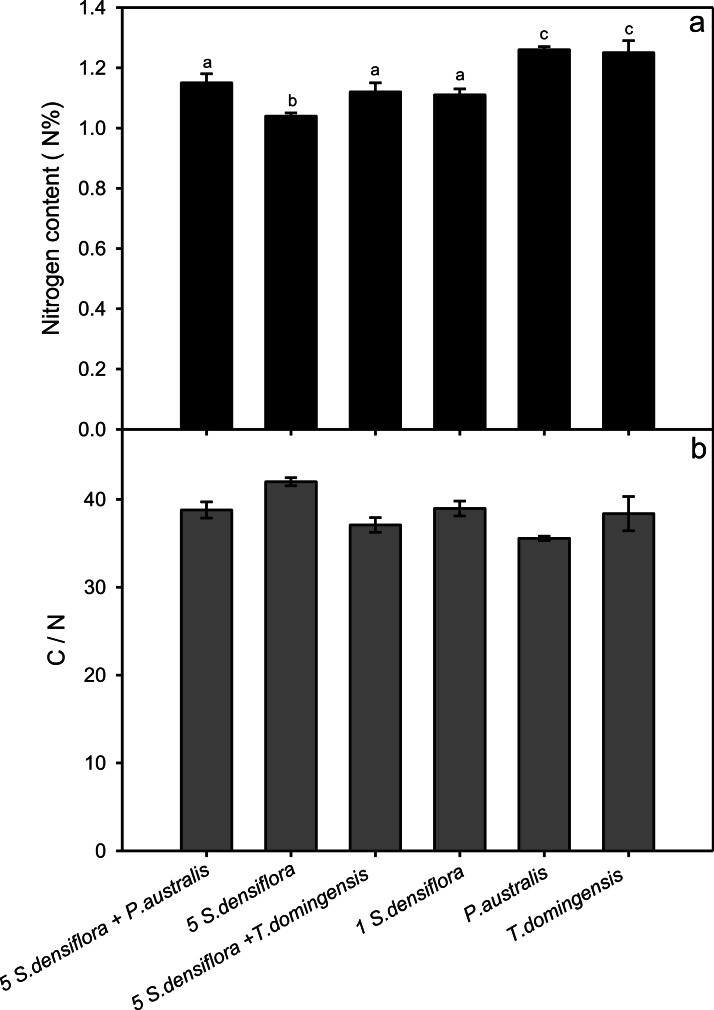
Nitrogen content and C:N ratio. (A) Nitrogen content (%), and (B) C: N ratio for *S. densiflora* in four different competition treatments (see Fig. 1) and for *Phragmites australis* and *Typha domingensis* adult stands. Different letters indicate significant difference among treatments (ANOVA, *P* < 0.05).

*Spartina densiflora* had lower leaf N content in all treatments compared with *P. australis* or *T. domingensis*. Leaf N content was lowest for *S. densiflora* seedlings growing in monoculture (1.04 ± 0.02% N), 1.11 ± 0.02%, for seedlings growing alone, 1.15 ± 0.03% for seedlings growing with *P. australis*, and 1.12 ± 0.03% for seedling growing with *T. domingensis*. The *P. australis* and *T. domingensis* only treatments had significantly higher leaf N content (ca. 1.25%) than all treatments with *S. densiflora* seedlings (*P* < 0.05; Fig. 3). As expected, C:N reflected leaf N content among the treatments (Fig. 3).

## Discussion

We hypothesized that mature native hydrophytes would be competitively superior under low salinity conditions and prevent the invasion of *S. densiflora*. *Phragmites australis* did effectively exclude *S. densiflora* seedlings, but *T. domingensis* stands were not able to completely stop establishment and growth. These differences in the survivorship and the growth of *S. densiflora* among treatments were likely related to contrasting biomass distribution patterns within the stands of the two native hydrophytes and not changes in the abiotic environment since soil characteristics and PPFD were similar among treatments. *P. australis* seemed to prevent the colonization of *S. densiflora* due to its very high biomass allocation to belowground structures, similar to that of *T. domingensis*, but showed more uniform occupation of the subterranean space than *T. domingensis*. *Phragmites australis* has a dense network of shallow rhizomes and roots, with corresponding high aboveground stem density, as opposed to the deep, sparse root structure of *T. domingensis* (JM Castillo, pers. obs., 2007). The more regular and dense occupation of the subterranean space just below the soil surface by *P. australis* may have prevented the establishment of *S. densiflora* seedlings, presumably by blocking the establishment of the subterranean rooting system. Empty space in the below-ground neighbourhood is often a key factor for plant establishment (McConnaugha & Bazzaz, 1991; Casper & Jackson, 1997). Nevertheless, Minchinton, Sympson & Bertness (2006) found that *P. australis* also effectively excluded other plant species by increased shoot and litter production rather than by changing soil properties or by below-ground competition.

*Spartina densiflora* did well in the early establishment phase when both native species were dormant and stem density was low, and although above-ground competition is negligible at this point (Engloner, 2009), the rapid decrease in growth over the growing season points to below-ground competition for limited space as the likely mechanism. *Spartina densiflora* has ruderal characteristics and readily colonizes bare substrates, but is less successful in well established plant communities. Castillo et al. (2008) found that *S. densiflora* invasion from seeds may be limited in Spanish marshes by inter-specific subterranean competition with the native *S. maritima* (Curtis). Similarly, tiller expansion of *S. densiflora* in North American marshes was higher in areas without native competitors (Kittelson & Boyd, 1997). *Phragmites australis* is a notoriously competitive hydrophyte in brackish and freshwater systems, and may effectively limit the spread of *S. densiflora* where they co-occur. Engels & Jensen (2010) described competitive displacement of *S. anglica* C.E. Hubbard by *P. australis* in North European freshwater marshes, and He et al. (2009) showed that established *P. australis* stands inhibited the development of *Suaeda salsa* (L) Pallas seedlings in China. The European *P. australis* lineage has invaded coastal and freshwater marshes throughout eastern North America (Saltonstall, 2002) where it has formed extensive monocultures and displaced diverse assemblages of native plants (Marks, Lapin & Randall, 1994; Tiner, 1997; Chambers, Meyerson & Saltonstall, 1999; Meyerson, Saltonstall & Windham, 2000), including native populations of *Typha* spp. (Chun & Choi, 2009), indigenous *P. australis* (Lambert, Dudley & Saltonstall, 2010), and *Spartina* spp. (Saltonstall, 2002; Robertson & Weis, 2005; Kimball & Able, 2007).

Nutrient levels and C:N were also generally consistent across treatments so competition for limiting nutrients did not appear to be a factor. Nitrogen content of seedlings was actually higher in the inter-specific competition treatments. We do not know from our study whether *P. australis* or *T. domingensis* captured or used nitrogen more efficiently, but other studies have shown that the growth of *P. australis* is stimulated by nitrogen which it efficiently convert to belowground biomass (Sillman & Bertness, 2004; Lambert, Dudley & Robbins, 2014). Rickey & Anderson (2004) found that *P. australis* displaced native *Spartina pectinata* Bosc. ex Link. under high nitrogen conditions.

In view of our results, management of invaded and susceptible marshes should focus on well-conserved communities of native hydrophytes as a way to passively resist invasion by *S. densiflora*. In some European marshes, *P. australis* has shown a general dye-back (Ostendorp, 1989) which, if it occurred in estuaries invaded by *S. densiflora* could open space for further invasion. In addition, planting *P. australis* in estuaries and river banks in areas already invaded by or susceptible to invasion by *S. densiflora* should be considered as viable option for creating a barrier to cordgrass expansion.

## Supplemental Information

10.7717/peerj.1260/supp-1 Supplemental Information 1 Manuscript data Raw data

## References

[ref-1] Byers JE, Goldwasser L (2001). Exposing the mechanisms and timing of impact of non indigenous species on native species. Ecology.

[ref-2] Casper BB, Jackson RB (1997). Plant competition underground. Annual Review of Ecology, Evolution, and Systematics.

[ref-3] Castillo JM, Figueroa E (2009). Effects of abiotic factors on the life span of the invasive cordgrass *Spartina densiflora* and the native *S. maritima* at low marshes. Aquatic Ecology.

[ref-4] Castillo JM, Mateos-Naranjo E, Nieva FJ, Figueroa E (2008). Plant zonation at salt marshes of the endangered cordgrass *Spartina maritima* invaded by *Spartina densiflora*. Hydrobiologia.

[ref-5] Chambers RM, Meyerson LA, Saltonstall K (1999). Expansion of *Phragmites australis* into tidal wetlands of North America. Aquatic Ecology.

[ref-6] Chun YM, Choi YD (2009). Expansion of *Phragmites australis* (Cav.) Trin. ex Steud. (*P. australis*) into *Typha* spp. (Cattail) Wetlands in Northwestern Indiana, USA. Journal of Plant Biology.

[ref-7] Crain CM, Bertness MD (2006). Ecosystem engineering across environmental gradients: implications for conservation and management. Bio. Science.

[ref-8] Curado G, Rubio-Casal AE, Figueroa E, Castillo JM (2010). Germination and establishment of the invasive cordgrass *Spartina densiflora* in acidic and metal polluted sediments of the Tinto river. Marine Pollution Bulletin.

[ref-9] Engels JG, Jensen K (2010). Role of biotic interactions and physical factors in determining the distribution of marsh species along an estuarine salinity gradient. Oikos.

[ref-10] Engels JG, Rink F, Jensen K (2011). Stress tolerance and biotic interactions determine plant zonation patterns in estuarine marshes during seedling emergence and early establishment. Journal of Ecology.

[ref-11] Engloner AI (2009). Structure, growth dynamics and biomass of reed (*Phragmites australis*)—a review. Flora.

[ref-12] He Q, Cui BS, Cai YZ, Deng JF, Sun T, Yang ZF (2009). What confines an annual plant to two separate zones along coastal topographic gradients?. Hydrobiologia.

[ref-13] Kimball ME, Able KW (2007). Nekton utilization of intertidal salt marsh creeks: tidal influences in natural *Spartina*, invasive *Phragmites*, and marshes treated for *Phragmites* removal. Journal of Experimental Marine Biology and Ecology.

[ref-14] Kittelson PM, Boyd MJ (1997). Mechanisms of expansion for an introduced species of cordgrass, *Spartina densiflora*, in Humboldt Bay, California. Estuaries.

[ref-15] Lambert AM, Dudley TL, Robbins J (2014). Nutrient enrichment and soil conditions drive productivity in the large-statured invasive grass *Arundo donax*. Aquatic Botany.

[ref-16] Lambert AM, Dudley TL, Saltonstall K (2010). Ecology and impacts of the large-statured invasive grasses *Arundo donax* and *Phragmites australis* in North America. Invasive Plant Science and Management.

[ref-17] Maestre FT, Callaway RM, Valladares F, Lortie CJ (2009). Refining the stress-gradient hypothesis for competition and facilitation in plant communities. Journal of Ecology.

[ref-18] Marks M, Lapin B, Randall J (1994). *Phragmites australis* (*Phragmites communis*): threats, management, and monitoring. Natural Areas Journal.

[ref-19] McConnaugha KDM, Bazzaz FA (1991). Is physical space a soil resource?. Ecology.

[ref-20] Meyerson LA, Saltonstall K, Windham L (2000). A comparison of *Phragmites australis* in freshwater and brackish marsh environments in North America. Wetlands Ecology and Management.

[ref-21] Minchinton TE, Sympson JC, Bertness MD (2006). Mechanisms of exclusion of native coastal marsh plants by an invasive grass. Journal of Ecology.

[ref-22] Nieva FJ, Díaz-Espejo A, Castellanos EM, Figueroa ME (2001). Field variability of invading populations of *Spartina densiflora* Brong. grown in different habitats of the Odiel marshes (SW Spain). Estuarine, Coastal and Shelf Science.

[ref-23] Ostendorp W (1989). Die-back of reeds in Europe: a critical review. Aquatic Botany.

[ref-24] Parker IM, Simberloff D, Lonsdale WM (1999). Impact: toward a framework for understanding the ecological effects of invaders. Biological Invasions.

[ref-25] Rhoades JD, Sparks DL, Page AL, Helmke PA, Loeppert RH (1996). Salinity: electrical conductivity and total dissolved solids. Methods of soil analysis. Part 3. Chemical methods.

[ref-26] Rickey MA, Anderson RC (2004). Effects of nitrogen addition on the invasive grass *Phragmites australis* and a native competitor *Spartina pectinata*. Journal of Applied Ecology.

[ref-27] Robertson TL, Weis JS (2005). A comparison of epifaunal communities associated with the stems of salt marsh grasses *Phragmites australis* and *Spartina alterniflora*. Wetlands.

[ref-28] Saltonstall K (2002). Cryptic invasion by a non-native genotype of the *P. australis*, *Phragmites australis*, into North America. Proceedings of the National Academy of Sciences of the United States of America.

[ref-29] Sharma P, Asaeda T, Kalibbala M, Fujino T (2008). Morphology, growth and carbohydrate storage of the plant *Typha angustifolia* at different water depths. Chemistry and Ecology.

[ref-30] Sillman BR, Bertness MD (2004). Shoreline development drives invasion of *Phragmites australis* and the loss of plant diversity on New England salt marshes. Conservation Biology.

[ref-31] Sobrero MT, Sabbatini MR, Fernandez OA (1997). Phenology and biomass dynamics of cattail (*Typha subulata*) in southern Argentina. Weed Science.

[ref-32] Tiner RW (1997). Managing *P. australis* (*Phragmites australis*) in Massachusetts: an overview of the species and control techniques.

[ref-33] Ungar IA (1998). Are biotic factors significant in influencing the distribution of halophytes in saline habitats?. Botanical Review.

